# Forbidden codon combinations in error-detecting circular codes

**DOI:** 10.1007/s12064-024-00431-6

**Published:** 2024-12-15

**Authors:** Elena Fimmel, Hadi Saleh, Lutz Strüngmann

**Affiliations:** Institute for Mathematical Biology Faculty of Computer Sciences, Mannheim University of Applied Sciences, 68163 Mannheim, Germany

**Keywords:** Genetic code, circular codes, codon usage, codon exclusion, translation process

## Abstract

Circular codes, which are considered as putative remnants of primaeval comma-free codes, have recently become a focal point of research. These codes constitute a secondary type of genetic code, primarily tasked with detecting and preserving the normal reading frame within protein-coding sequences. The identification of a universal code present across various species has sparked numerous theoretical and experimental inquiries. Among these, the exploration of the class of 216 self-complementary $C^3$-codes of maximum size 20 has garnered significant attention. However, the origin of the number 216 lacks a satisfactory explanation, and the mathematical construction of these codes remains elusive. This paper introduces a new software designed to facilitate the construction of self-complementary $C^3$-codes (of maximum size). The approach involves a systematic exclusion of codons, guided by two fundamental mathematical theorems. These theorems demonstrate how codons can be automatically excluded from consideration when imposing requirements such as self-complementarity, circularity or maximality. By leveraging these theorems, our software provides a novel and efficient means to construct these intriguing circular codes, shedding light on their mathematical foundations and contributing to a deeper understanding of their biological significance.

## Introduction

Genomes are dynamic units that change over time through more or less minor modifications to the nucleotide sequence. These heritable changes and errors in the nucleotide sequence of DNA are called mutations. Mutations are unavoidable and a necessary basis of evolution, but they usually have a negative effect on the organism. To limit the damage, organisms have developed effective repair mechanisms that limit the frequency of mutations to a tolerable level. Insertions or deletions of individual nucleotides account for almost a quarter of all gene mutations in the human genome (Abecasis et al. 2010). If they occur in the exon region of a gene, they lead to a shift in the reading frame (frameshift mutation) so that the wrong amino acids are used to synthesise the protein from the lesion onwards during translation. The shift often also results in a stop codon, so that translation stops shortly after the lesion (Seligmann and Pollock 2004).

The question of how frameshift mutations in particular are recognised has occupied biologists since the 1950 s (Watson and Crick 1953; Shepherd 1981). Crick proposed the hypothesis that the coding codons only form a subset of all codons, so that the frameshifts are recognised immediately (Crick et al. 1957). He called such subsets (codes) comma-free codes. After the Poly-U experiment Nirenberg and Matthaei (1961) at the latest, it became clear that Crick’s beautiful hypothesis had been experimentally disproved. As a reminder: According to Crick’s hypothesis, only codons of a comma-free code should be able to encode amino acids, while the codon *UUU* cannot be contained in a comma-free code, as will be explained later in the article. As a result, the theory of comma-free codes, as well as other classes of codes that can recognise frameshift, was only sporadically pursued further (see, for instance, Golomb and Gordon 1965) for a long time until the mid-1990 s, when another class of codes which could be responsible for frameshift recognition was identified purely statistically in coding sequences by Arquès and Michel (1996), namely the class of self-complementary $C^3$ codes. In recent years, this topic has been actively pursued, so that some construction and functional principles of circular codes have been uncovered (Fimmel and Strüngmann 2016, 2015; Fimmel et al. 2016; Fimmel and Strüngmann 2016; Fimmel et al. 2017 Oct). In particular, the question of forbidden combinations of two, three or four codons in self-complementary $C^3$-codes has already been investigated in Bussoli et al. (2011). Computer tools have also been developed (Fimmel et al. 2017) to make it easier to deal with the huge number of combinatorial possibilities and to facilitate pattern searches.

In this article, we address the question of which codon combinations are impossible in different classes of error-detecting codes. The aim is to better understand the construction principles of such codes. For this purpose, a computer aid tool was developed (https://www.cammbio.hs-mannheim.de/research/software/codonpairselector.html), which makes it easier to recognise patterns and then underlay them mathematically. The following classes of codes were analysed: comma-free codes, self-complementary circular codes and self-complementary $C^3$-codes. It is also shown that not every self-complementary $C^3$-code can be extended to a self-complementary $C^3$-code of maximum size (Sect. 5.1). It has been known for several years (Bussilo et al. 2012) that there are exactly 216 self-complementary $C^3$-codes of maximum size. The number could not be explained; until now, they were calculated with computer help. In this article, we give an explanation for this number (Sect. 5.2).

## Definitions and notations

We will denote the genetic alphabet, which consists of four letters (nucleotides), as

$$\mathcal {B}:=\{U(T), C, A, G\}.$$

The four letters of the alphabet stand for *Uracil (Thymine), Cytosine, Adenine* and *Guanine*, in short *U*(*T*), *C*, *A*  and *G*. Three-letter words (called *codons*) over $\mathcal {B}$ encode individual amino acids and stop signals. Mathematically, they are elements of the space $\mathcal {B}^3$. In the following, we will analyse the construction of some subsets of $\mathcal {B}^3$ that are likely to be responsible for frame maintenance during the translation process. Already in the 1950 s, such subsets were considered by Crick et al as a potential answer to the frameshift problem (Crick et al. 1957). Crick called them comma-free codes:

### Definition 1

A trinucleotide code $X \subseteq \mathcal {B}^3$ is called *comma-free* if for any given two codons $x_1, x_2\in X$ any subcodon of the concatenation $x_1 x_2$ except $x_1, x_2$ themselves does not belong to *X*. We will call a trinucleotide comma-free code *X* *of maximum size* if it contains exactly 20 codons.

**Fig. 1:**
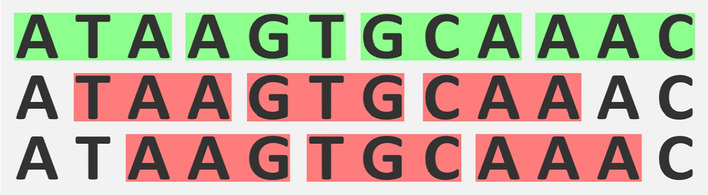
For comma-free codes, a frameshift is detected immediately. All codons highlighted in red in the second and the third row are not in the comma-free code $\{ATA,AGT,GCA,AAC\}$

Being comma-free means that a frameshift of one or two bases is immediately detected in the reading process (see Fig. 1).

It is immediately clear that the periodic codons *AAA*, *CCC*, *GGG*, *UUU*(*TTT*) cannot be contained in a comma-free code, as the frameshift cannot be recognised in a sequence containing only them. For example, the frameshift played no role in the famous Poly-U experiment by Nirenberg and Matthaei 1961, as the sequence consisted only of *U* and the sequence of translated amino acids contained only phenylalanine. It is also obvious that two cyclically shifted codons cannot be together in a comma-free code. For example, let us look at the codon *CTG*:

$$\begin{aligned} CTGCTGCTGCTG... \end{aligned}$$

$$\begin{aligned} C{TGC}TGCTGCTG... \end{aligned}$$

$$\begin{aligned} CT{GCT}GCTGCTG... \end{aligned}$$

Thus, any comma-free code can at most contain one codon out of the three *CTG*, *TGC* and *GCT* and similarly for any other codon. These simple observations allow us to estimate the maximum size of a comma-free code:

$$20=\frac{64-4}{3}.$$

Interestingly, it is also the actual maximum size of such codes. The complete list of comma-free codes of maximum size was already calculated by Golomb et al. (1958) end of the 1950 s and contains a total of 408 such codes.

Let us describe the cyclic shift operators formally. In the following, we denote them $\alpha _1:\mathcal {B}^3\rightarrow \mathcal {B}^3$ and $\alpha _2:\mathcal {B}^3\rightarrow \mathcal {B}^3$ and they act as follows

$$\alpha _1(N_1N_2N_3)=N_2N_3N_1\quad \text{ and }\quad \alpha _2(N_1N_2N_3)=N_3N_1N_2$$

for any codon $N_1N_2N_3 \in \mathcal {B}^3$ (see for instance Fimmel et al. 2015). Another permutation of the positions in a codon, the so-called *reversing permutation* which reverses a codon, i.e. $\overleftarrow{{N_1N_2N_3}}:=N_3N_2N_1$, with $N_i\in \mathcal {B}$, plays an important role in the biological context.

After Crick’s hypothesis was experimentally disproved, another class of error-detecting codes, statistically identified in genomes across species in the mid-1990 s (Arquès and Michel 1996), has generated renewed interest. These are the so-called circular codes. Let us first formally define what the property of circularity means:

### Definition 2

We will call a set of codons $X\subseteq \mathcal {B}^3$ a *trinucleotide circular code* if any word over the alphabet $\mathcal {B}$ written on a circle has at most one decomposition into words from *X*. By word written on a circle, it is intended that after the last letter the word starts again (from its first letter). We will call a trinucleotide circular code *X* *of maximum size* if it contains exactly 20 codons.

**Fig. 2:**
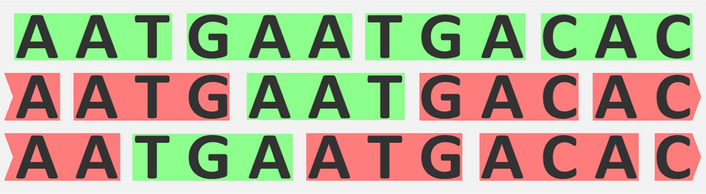
For circular codes, a frameshift is at most detected after a few codons. The codons highlighted in red in the second and third row are not in the circular code $\{AAT, GAA, TGA, CAC\}$

Circular codes do not allow the detection of a frameshift immediately as comma-free codes do but eventually after a few codons (see Fig. 2). Thus, it is obvious that any comma-free code is also circular.

The code that Arquès and Michel (1996) statistically identified in genomes of all species is

$$X_{0}=\left\{ { \begin{array}{cccccccc} AAC,& AAT,& ACC,& ATC,& ATT,& CAG,& CTC,& CTG,\\ GAA,& GAC,& GAG,& GAT,& GCC,& GGC,& GGT,& GTA,\\ GTC,& GTT,& TAC,& TTC \ \end{array}}\right\} .$$

This code $X_0$ has some other very strong properties in addition to the property of circularity. First, not only the code itself is circular, but also the two codes that are obtained from it through circular shifts. This allows frameshift detection also in frames one and two:

### Definition 3

Let $X\subseteq \mathcal {B}^3$. We will say that *X* is a $C^3$* code* if *X*, as well as $X_1$ and $X_2$ are circular, where $X_1:=\alpha _1(X)$ and $X_2:=\alpha _2 (X)$.

Studies in recent years (Fimmel et al. 2015) have shown that the group of all bijective mappings of the alphabet $\mathcal {B}$ in itself, the so-called symmetric group, plays an important role in preserving the property of circularity (and in particular comma-freeness and $C^3$ property). Formally, the symmetric group on the set $\mathcal {B}$ is defined as

$$\textbf{S}_{\mathcal {B}}= \{ \varvec{\pi }: \mathcal {B}\rightarrow \mathcal {B}\mid \pi \textit{ is bijective} \}$$

with the group operation of function composition. Bijective mappings $\pi :\mathcal {B}\rightarrow \mathcal {B}$ can be applied componentwise to $x\in \mathcal {B}^3$ and thus induce a bijective map $\mathcal {B}^3\rightarrow \mathcal {B}^3$, which we will denote also by $\pi$. Hence $\pi$ systematically exchanges bases in a codon or sequence of codons and there are exactly 24 such transformations. A very important mapping from $S_{\mathcal {B}}$ is the so-called *complementarity mapping*:

$$\textbf{c}: \mathcal {B}\rightarrow \mathcal {B}$$

with

$$\mathbf{c(A)= T, c(T)=A, c(C)=G, c(G)=C.}$$

which assigns to each basis its complementary basis. As already mentioned above, all mappings from $S_{\mathcal {B}}$ preserve the property of circularity (see Fimmel et al. 2015):

2.1 $$\begin{aligned} \textit{ Any permutation from }S_{\mathcal {B}} \textit{ preserves comma-freeness and circularity, hence the }C^3\textit{ property} \end{aligned}$$

Furthermore, along with each codon, the code $X_0$ also contains the corresponding anticodon. This property is called self-complementarity:

### Definition 4

Let $X\subseteq \mathcal {B}^3$. We will call *X* *self-complementary* if with each codon $x\in X$ $\overleftarrow{{c(x)}}$ is also in *X*.

Again computer calculations showed that there are exactly 528 self-complementary circular codes of maximum size (Michel and Pirillo 2010). This class contains the class of 216 self-complementary $C^3$ codes of maximum size (see Bussilo et al. 2012; Michel et al. 2016) as a subclass.

However, only eight of the bijective mappings from $S_{\mathcal {B}}$, namely those commuting with the mapping *c*, preserve the property of self-complementarity. These maps were identified in Fimmel et al. (2015); Fayazi et al. (2021) and form a subgroup of $S_{\mathcal {B}}$ that is isomorphic to the so-called dihedral group. In the following, we denote this subgroup $\textbf{L}$:

2.2 $$\begin{aligned} \textit{ If }X\textit{ is a self-complementary circular code and} \pi \in L,\textit{ then the code }\pi {(X)} \end{aligned}$$

*is circular and self-complementary as well.*

Finally, the *reversing permutation* also preserves self-complementarity and circularity (see Fimmel et al. 2015). Thus, we have

2.3 $$\begin{aligned} \textit{ If }X\textit{ is a circular (comma-free, }C^3\textit{-) code, then its reversed code }\overleftarrow{{X}} \end{aligned}$$

* is circular (comma-free, *$C^3$*-) as well;*

2.4 $$\begin{aligned} \textit{ If }X\textit{ is a self-complementary code, then its reversed code }\overleftarrow{{X}} \end{aligned}$$

*is self-complementary as well.*

## A novel software tool "CodonPairSelector"

CodonPairSelector is a tool we created to find patterns in exclusion rules between codons based on comma-freeness, circularity, self-complementarity, maximality or the $C^3$-property. The tool can be accessed via the link https://www.cammbio.hs-mannheim.de/research/software/codonpairselector.html

The tool allows the user to progressively build a code from individual codons and immediately check which other codons cannot be included in this code without breaking circularity (respectively, comma-freeness or the $C^3$-property). While the tool cannot be used to find every instance of and verify an exclusion rule, it can be used as a starting point to create hypothesis about these rules, which can later be algorithmically verified. It also helps in finding counterexamples that would otherwise be difficult to find. For example, it was used to construct a counterexample to the hypothesis that every $C^3$-code can be extended to a $C^3$-code of maximum size (compare Sect. 5.1).

### Description of features

After the user selects a set of codons, the programme adds one new codon to the set and checks if this new set is circular (respectively, comma-free or has the $C^3$-property). If it is not, the new codon is highlighted as excluded, and the same process is repeated for every other codon. The programme checks for circularity (respectively, comma-freeness or the $C^3$-property) by either running a graph-theoretic based algorithm (see Fimmel et al. 2016 for the graph theory) or checking against a database of all self-complementary circular codes ($C^3$-codes), respectively, comma-free codes of maximum size.

**Fig. 3:**
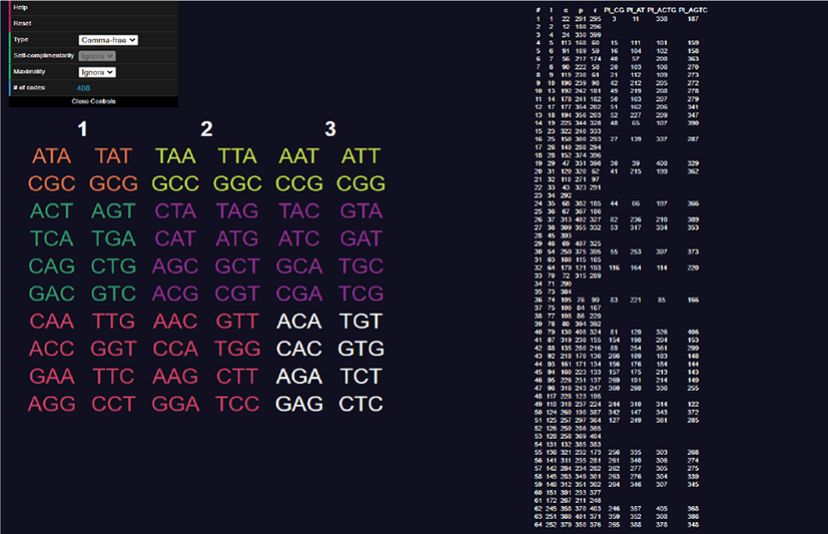
Screenshot of the tool **CodonPairSelector**. Codons are coloured orange for type 0, yellow for type 1, green for type 2, purple for type 3, red for type 4 and white for type 5. No codons are selected. The list of all maximal comma-free codes is displayed on the right side

**Fig. 4:**
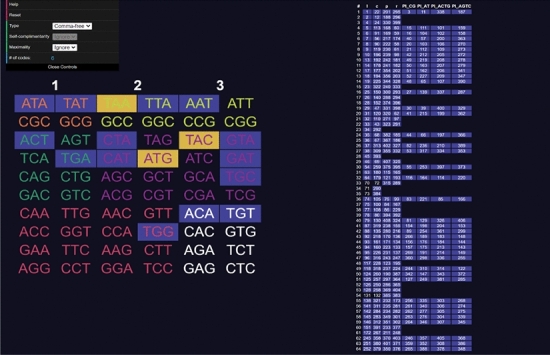
Screenshot of the tool **CodonPairSelector**. Various codons are selected. The list of all maximal comma-free codes is displayed on the right side

**Fig. 5:**
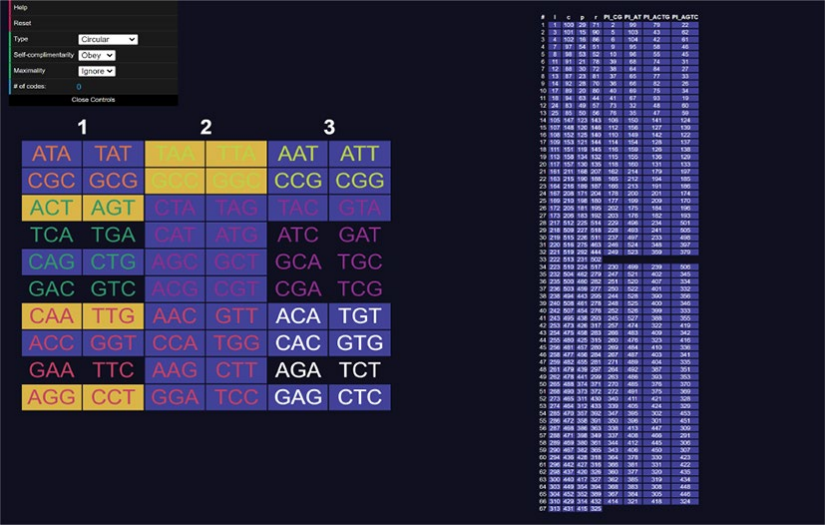
Screenshot of the tool **CodonPairSelector**. Various codons are selected. The list of all self-complementary maximal circular codes is displayed on the right side. All the codes in the list are highlighted as the codons cannot be extended to be of maximum size

**Fig. 6:**
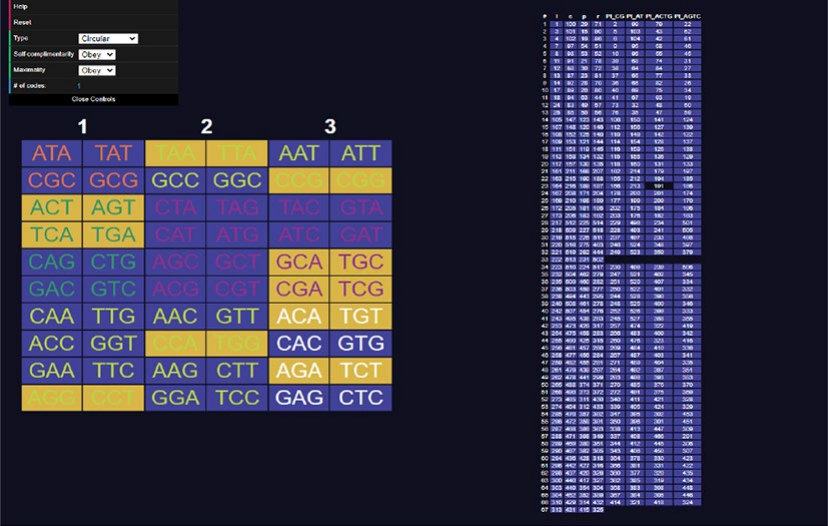
Screenshot of the tool **CodonPairSelector**. All the codons from a code (191) are selected. The list of all self-complementary maximal circular codes is displayed on the right side

Opening the website, a user will be presented with a table of codons on the left side and a table of codes on the right (see Fig. 3). The codons can be clicked to select and deselect them, adding them to the code being constructed. Added codons are highlighted in yellow. As codons are added, other codons will be highlighted in blue indicating they are excluded using the current rule (comma-free, circular or $C^3$-property) (see Fig. 4). On the right side table, codes that do *not* include all the codons selected by the user are highlighted in blue. Non-highlighted codes contain every codon selected by the user. Sometimes all codes can be highlighted, meaning the currently selected set of codons cannot be extended to a code of maximum size or is not self-complementary (labelled non-self-complementary codes are too numerous to show on the website) (see Fig. 5). Clicking on a code in the right side table will highlight all the codons included in it (see Fig. 6).

On the top left is a menu with various options.

- "**Help**" links to the GitHub repository.
- "**Reset**" deselects all codons starting the construction process over.
- "**Copy current code to clipboard**" copies the selected codons to clipboard as a comma-separated list.
- "**Import code**" prompts the user to type in a list of comma-separated codons and selects those in the tool.
- "**Export table**" downloads a.json file of the entire right side table for using as data or checking the codons of every code.
- "**Type**" tells the programme which type of code is being tested for (comma-free, circular or $C^3$-property). It also changes which codes of maximum size are displayed on the right side.
- "**Self-complementarity**" determines whether or not self-complementary should be enforced, most relevant for circular codes.
- "**Maximality**" tells the programme if it should exclude codons not only for breaking circularity, but also if they prevent the code from reaching a maximal number of codons.
- "**# of codes**" shows how many codes of maximum size contain the set of selected codons (the number of unhighlighted codes from the right side table).

Codons are colour coded depending on their type: orange for type 0, yellow for type 1, green for type 2, purple for type 3, red for type 4 and white for type 5. For this classification see Definition 4.2 the next section.

## Forbidden codon combinations in self-complementary error-detecting codes (of maximum size)

In this section, we will mainly consider two classes of error-detecting and error-correcting codes that have appeared in the development and theory of the genetic code: the class $\mathcal {CIRC}$ of all self-complementary circular codes, and the class $\mathcal {C}3$ of all self-complementary $C^3$ codes. We are interested in determining which codon combinations are impossible in such codes. It is clear that with a codon $x\in X\in \mathcal {CIRC} (\mathcal {C}3$) its circular shifts $\alpha _1(c)$ and $\alpha _2(c)$ are excluded. It was also noted earlier (see Fimmel et al. 2015; Fimmel and Strüngmann 2016) that codons of the form $c(N)Nc(N), N\in \mathcal {B}$ can never occur in a self-complementary circular code. However, it turns out that there are other additional constraints. Since we are dealing with self-complementary codes, it is reasonable that these constraints involve codon/anticodon pairs rather than single codons. We will consider the following codon types:

### Definition 5

Given a codon *x* in $\mathcal {B}^3$ we classify it according to the following types which refer to the structure of *x*:

**Type 0: ** $Nc(N)N, N\in \mathcal {B}$ This class includes the codon/anticodon pairs *ATA*/*TAT*, *CGC*/*GCG*
**Type 1: ** *NNc*(*N*) or $c(N)NN, N\in \mathcal {B}$. This class includes the codon/anticodon pairs
  $$AAT/ATT, TAA/TTA, GCC/GGC, CCG/CGG.$$
**Type 2: ** $N_1N_2c(N_1), N_1,N_2\in \mathcal {B}, N_1\ne N_2, N_2\ne c(N_1)$ This class includes the codon/anticodon pairs
  $$ACT/AGT, TCA/TGA, CAG/CTG, GAC/GTC.$$
**Type 3: ** $N_2N_1c(N_1)$ or $N_1c(N_1)N_2,\quad N_1,N_2\in \mathcal {B}, N_1\ne N_2, N_2\ne c(N_1)$ This class includes the codon/anticodon pairs
  $$CTA/TAG, CAT/ATG, AGC/GCT, ACG/CGT,$$
  $$TAC/GTA, ATC/GAT, GCA/TGC, CGA/TGC,$$
  and
**Type 4: ** $N_1N_1N_2$ or $N_2N_1N_1,\quad N_1\ne N_2, N_2\ne c(N_1)$. This class includes the codon/anticodon pairs
  $$AAC/GTT, AAG/CTT, CCA/TGG, GGA/TCC$$
  $$ACC/GGT, AGG/CCT, CAA/TTG, GAA/TTC.$$
**Type 5: ** $N_1N_2N_1,\quad N_1\ne N_2, N_2\ne c(N_1)$. This class includes the codon/anticodon pairs
  $$ACA/TGT, AGA/TCT, CAC/GTG, GAG/CTC.$$

**Fig. 7:**
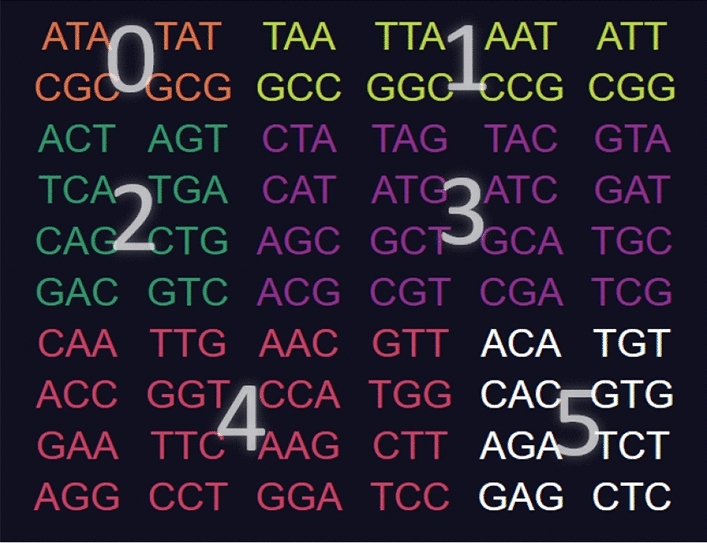
Screenshot of the **CodonSelector** software where the coloured codon/anticodon pairs are marked with their numbers according to Definition 5

In order to visualise the different types defined in Definition 5, the reader can find a screenshot of the **CodonSelector** software in Fig. 7 where the coloured codon/anticodon pairs are marked with their type number

Firstly, we make a useful observation:

### Lemma 1

Let $x \in \mathcal {B}^3$ be any codon of type n (with $n \in \{0, \cdots , 5\}$) and $\pi \in L$ be any permutation from the group *L*.

1. Then $\pi (x)$ is again of type *n*.
2. The reversed codon $\overleftarrow{{x}}$ is again of type *n*.

### Proof

1. This is clear since the definition of types only involves the complementary permutation *c* and *L* consists precisely of all permutations that commute with *c*.
2. Direct verification (see Definition 5).

In the next subsections, we will start investigating how codon/anticodon pairs of a certain type exclude other codon/anticodon pairs for certain classes of codes.

### The class $\mathcal {CIRC}$

We now focus on the class $\mathcal {CIRC}$ of self-complementary circular codes and start with an easy observation.

#### Lemma 2

Let $c_1, c_2\in \mathcal {B}^3$ and $\pi \in L$ be any permutation from the group *L*.

1. If $c_1$ and $c_2$ cannot appear in a circular code together then this is also true for $\pi {(c_1)}$ and $\pi {(c_2)}$.
2. If $c_1$ and $c_2$ cannot appear in a circular code together then this is also true for $\overleftarrow{{c_1}}$ and $\overleftarrow{{c_2}}$.

#### Proof

Assume that $\overleftarrow{{c_1}},\overleftarrow{{c_2}}\in Y\quad (\pi {(c_1)},\pi {(c_2)}\in Y)$ where *Y* is a circular code. According to Fimmel et al. (2015) $\overleftarrow{{Y}} \quad (\pi ^{-1}{(Y)})$ is also a circular code and $c_1, c_2\in \overleftarrow{{Y}} \quad (c_1, c_2\in \pi ^{-1}{(Y)})$. This is a contradiction to the circularity of $\overleftarrow{{Y}} \quad (\pi ^{-1}{(Y)})$.

We now show that a codon/anticodon pair of one of the 6 classes defined above in a self-complementary circular code excludes its circular shifts but also other codon/anticodon pairs of the same or a different type. The above lemma 2 is very helpful in reducing the number of cases to be considered. For example, in the theorems below for codon types 3 or 4, which each contains two differently structured codon types that are reverse to each other, proof is given for only one of them.

#### Theorem 1

Let $X\in \mathcal {CIRC}$ be a self-complementary circular code. Then the following statements are true:

1. A codon/anticodon pair *Nc*(*N*)*N*/*c*(*N*)*Nc*(*N*) of type 0 cannot be in *X*.
2. A codon/anticodon pair $N_1N_1c(N_1)/N_1c(N_1)c(N_1)$ of type 1 with $N_1\in \mathcal {B}$ cannot be in a self-complementary circular code together with its cyclic shifts and the two codon/anticodon pairs
  $$c(N_2)N_2N_1, \quad c(N_1)c(N_2)N_2\notin X,\quad N_2\ne N_1, N_2\ne c(N_1)$$
  of type 3 (for $N_2$ there are two choices). For all combinations with the other codon/anticodon pairs, there is a self-complementary circular code that contains them.
3. A type 2 or type 4 codon/anticodon pair cannot be in a self-complementary circular code together with its cyclic shifts. For all combinations with the other codon/anticodon pairs, there is a self-complementary circular code that contains them.
4. A codon/anticodon pair $N_1N_2c(N_2)/N_2c(N_2)c(N_1)\in X$ of type 3 cannot be in a self-complementary circular code together with its cyclic shifts, the type 1 codon identified in (1) and its reversed codon/anticodon pair
  $$c(N_2)N_2N_1/c(N_1)c(N_2)N_2\notin X$$
  and
  $$c(N_2)c(N_1)N_1/c(N_1)N_1N_2\notin X$$
  (both of type 3). For all combinations with the other codon/anticodon pairs, there is a self-complementary circular code that contains them.
5. A codon/anticodon pair $N_1N_2N_1/c(N_1)c(N_2)c(N_2)\in X$ of type 5 cannot be in a self-complementary circular together with its cyclic shifts and another codon/anticodon pair of type 5, namely $N_2N_1N_2/c(N_2)c(N_1)c(N_1)\notin X$. For all combinations with the other codon/anticodon pairs, there is a self-complementary circular code that contains them.

#### Proof

We will prove in all the statements that the given codon/anticodon pairs exclude the mentioned codon/anticodon pairs whereas this is clear for the cyclic shifts. However, all the converse implications, i.e. that these are the only codon/anticodon pairs that are excluded, follow from computer calculations using the new tool CodonPairSelector.

1. Assume that $Nc(N)N/c(N)Nc(N)\in X$. Let us now consider the concatenation
  $$Nc(N)Nc(N)Nc(N).$$
  It has two decompositions on a circle
  $$Nc(N)N|c(N)Nc(N)\quad \text{ and }\quad c(N)Nc(N)|Nc(N)N.$$
2. Assume that
  $$N_1N_1c(N_1)/N_1c(N_1)c(N_1)\in X$$
  as well as
  $$c(N_2)N_2N_1, c(N_1)c(N_2)N_2\in X.$$
  Let us now consider the concatenation
  $$c(N_1)c(N_2)N_2N_1N_1c(N_1).$$
  It has two decompositions on a circle
  $$c(N_1)c(N_2)N_2|N_1N_1c(N_1)\quad \text{ and }\quad c(N_2)N_2N_1|N_1c(N_1)c(N_1).$$
  This is a contradiction to the circularity of *X*.
3. Obvious.
4. Assume that
  $$N_1N_2c(N_2)/N_2c(N_2)c(N_1)\in X$$
  as well as
  $$c(N_2)N_2N_1/c(N_1)c(N_2)N_2\in X.$$
  Consider
  $$N_1N_2c(N_2)c(N_1)c(N_2)N_2$$
  with two decompositions on a circle
  $$N_1N_2c(N_2)|c(N_1)c(N_2)N_2\quad \text{ and }\quad N_2c(N_2)c(N_1)|c(N_2)N_2N_1.$$
  It is a contradiction to the circularity of *X*. Assume that
  $$N_1N_2c(N_2)/N_2c(N_2)c(N_1)\in X$$
  as well as
  $$c(N_2)c(N_1)N_1/c(N_1)N_1N_2\in X.$$
  Consider
  $$N_1N_2c(N_2)c(N_1)N_1N_2c(N_2)c(N_1)N_1N_2c(N_2)c(N_1)$$
  with two decompositions on a circle
  $$N_1N_2c(N_2)|c(N_1)N_1N_2|c(N_2)c(N_1)N_1|N_2c(N_2)c(N_1)$$
  and
  $$N_2c(N_2)c(N_1)|N_1N_2c(N_2)|c(N_1)N_1N_2|c(N_2)c(N_1)N_1.$$
  It is a contradiction to the circularity of *X*.
5. Assume that
  $$N_1N_2N_1/c(N_1)c(N_2)c(N_1)\in X$$
  as well as
  $$N_2N_1N_2/c(N_2)c(N_1)c(N_2)\in X.$$
  Let us now consider the concatenation
  $$N_1N_2N_1N_2N_1N_2.$$
  It has two decompositions on a circle
  $$N_1N_2N_1|N_2N_1N_2\quad \text{ and }\quad N_2N_1N_2|N_1N_2N_1.$$
  This is a contradiction to the circularity of *X*.

We now pass to self-complementary $C^3$-codes in the next section.

### The class $\mathcal {C}3$

We now consider the class $\mathcal {C}^3$ of all self-complementary $C^3$-codes, and it turns out that surprisingly the $C^3$-property does not substantially force more codons to be excluded except for one case of codons of type 4.

#### Theorem 2

Let $X\in \mathcal {C}^3$ be a self-complementary $C^3$-code. Then the following statements are true:

1. A codon/anticodon pair of type 0 *Nc*(*N*)*N*/*c*(*N*)*Nc*(*N*) cannot be in *X*.
2. A codon/anticodon pair of type 1 $N_1N_1c(N_1)/N_1c(N_1)c(N_1)$ with $N_1\in \mathcal {B}$ only excludes^1^, apart from its cyclic shifts the two codon/anticodon pairs
  $$c(N_2)N_2N_1, \quad c(N_1)c(N_2)N_2\notin X,\quad N_2\ne N_1, N_2\ne c(N_1)$$
  of type 3 (for $N_2$ there are two choices).
3. A type 2 codon/anticodon pair only excludes its cyclic shifts.
4. A codon/anticodon pair of type 3 $N_1N_2c(N_2)/N_2c(N_2)c(N_1)\in X$ excludes, apart from its cyclic shifts and the type 1 codon identified in 2), also its reversed codon/anticodon pair
  $$c(N_2)N_2N_1/c(N_1)c(N_2)N_2\notin X$$
  and
  $$c(N_2)c(N_1)N_1/c(N_1)N_1N_2\notin X$$
  (both of type 3).
5. A codon/anticodon pair of type 5 $N_1N_2N_1/c(N_1)c(N_2)c(N_2)\in X$ excludes, apart from its cyclic shifts, another codon/anticodon pair of type 5, namely $N_2N_1N_2/c(N_2)c(N_1)c(N_1)\notin X$.
6. A type 4 codon/anticodon pair $N_1N_2N_2/c(N_2)c(N_2)c(N_1)$, respectively, $N_1N_1N_2/c(N_2)c(N_1)c(N_1)$, excludes its cyclic shifts and the corresponding type 4 codon/anticodon pair $N_2N_1N_1/c(N_1)c(N_1)c(N_2)$, respectively, $N_2N_2N_1/c(N_1)c(N_2)c(N_2)$.

#### Proof

Statements (1), (2), (3), (4) and (5) follow directly from Theorem 1 since a $C^3$-code is also circular. We only need to prove (6) and also here only the second part since it is clear that cyclic shifts are excluded. Thus assume that a type 4 codon/anticodon pair $N_1N_2N_2/c(N_2)c(N_2)c(N_1)$ as well as

$$N_2N_1N_1/c(N_1)c(N_1)c(N_2)$$

are in *X*. Then a shift 2 yields that $N_2N_1N_2$ and $N_1N_2N_1$ are in $\alpha _2(X)$ which contradicts circularity of $\alpha _2(X)$ since *X* is a $C^3$-code.

If we compare Theorem 2 with Theorem 1, we see that the only difference is point 5, which states that a codon/anticodon pair of type 4 excludes, apart from its cyclic shifts, another codon/anticodon pair of type 4.

In the next section, we will consider the class of comma-free codes.

### The class $\mathcal {COM}$

We now turn our attention to the class $\mathcal {COM}$ of comma-free codes and drop the assumption of self-complementarity.

#### Theorem 3

Let $X\in \mathcal {COM}$ be a comma-free code. Then the following statements are true:

1. A type 0 codon $N_1c(N_1)N_1\in X$ with $N_1\in \mathcal {B}$ excludes, apart from its cyclic shifts, only its anticodon and 4 further codons of type 3:
  $$c(N_1)N_1c(N_1)\notin X$$
  as well as
  $$c(N_1)N_1N_2, \quad N_2N_1c(N_1)\notin X,\quad N_2\ne N_1, N_2\ne c(N_1)$$
2. A type 1 or type 4 codon only excludes its cyclic shifts.
3. A type 2 codon $N_1N_2c(N_1)\in X$ excludes, apart from its cyclic shifts, only the two codons of type 5
  $$N_2N_1N_2,\quad N_2c(N_1)N_2\notin X, \quad N_2\ne N_1, N_2\ne c(N_1).$$
4. A type 3 codon $N_2N_1c(N_1)\in X$ excludes, apart from its cyclic shifts and a codon of type 0 $N_1c(N_1)N_1$ (see 1), only a codon of type 5
  $$N_1N_2N_1\notin X, \quad N_2\ne N_1, N_2\ne c(N_1).$$
5. A type 5 codon $N_1N_2N_1\in X,\quad N_2\ne N_1, N_2\ne c(N_1)$ excludes, apart from its cyclic shifts, one further codon of type 5, namely $N_2N_1N_2\notin X$, two codons of type 2 $N_2N_1c(N_2), \quad c(N_2)N_1N_2\notin X$ and two codons of type 3 $N_2N_1c(N_1), \quad c(N_1)N_1N_2\notin X$

#### Proof

As in the proofs before we only show that the given codon excludes its cyclic shifts and the stated codons. The fact that these are all excluded codons follows from computer calculations using the software CodonPairSelector.

1. Consider the concatenation $N_1c(N_1)N_1c(N_1)N_1c(N_1)$. It contains $N_1c(N_1)N_1$ as a subcodon. Consider the concatenation $N_1c(N_1)N_1c(N_1)N_1N_2$. It contains $N_1c(N_1)N_2$ as a subcodon. Consider the concatenation $N_2N_1c(N_1)N_1c(N_1)N_1)$. It contains $N_1c(N_1)N_2$ as a subcodon.
2. Obvious.
3. Consider $N_2N_1N_2N_1N_2c(N_1)$. It contains $N_2N_1N_2$ as a subcodon. Consider $N_1N_2c(N_1)N_2c(N_1)N_2$. It contains $N_2c(N_1)N_2$ as a subcodon.
4. Consider $N_1N_2N_1N_2N_1c(N_1)$. It contains $N_1N_2N_1$ as a subcodon.
5. Consider $N_1N_2N_1N_2N_1N_2$. It contains $N_1N_2N_1$ as a subcodon. Consider $N_1N_2N_1N_2N_1c(N_2)$. It contains $N_1N_2N_1$ as a subcodon. Consider $c(N_2)N_1N_2N_1N_2N_1$. It contains $N_1N_2N_1$ as a subcodon. Consider $c(N_1)N_1N_2N_1N_2N_1$. It contains $N_1N_2N_1$ as a subcodon.

The above theorem shows that the inclusion of codons of type 0 or 5 in a comma-free code imposes the most restrictions. With such a codon, 7 additional codons are excluded from the code.

### Forbidden codon combinations involving more than two codon/anticodon pairs.

All the rules established so far have involved two codon/anticodon pairs. The inclusion of the first in a self-complementary circular code excludes the second, but importantly all these rules are symmetrical, meaning the inclusion of the second excludes the first. In this section, we will discuss every rule in which two codon/anticodon pairs exclude a third pair. These rules are similarly "symmetrical", meaning that including any two of the three pairs in a code excludes the final pair. Furthermore, certain proofs require the presence of all 6 codons from all 3 pairs in the code, so in a non-self-complementary code we could include up to 5 of the 6 codons without necessarily breaking circularity. This also means that the anticodons are sometimes, but not always required for the proof.

With this in mind, we will no longer speak of codons or pairs excluding others, but rather of a set of codons from which all but one can be included in a circular code.

#### Theorem 4

Let *X* be a circular code. Then the following statements are true:

1. *X* can include a maximum of 2 out of 3 codons from the set
  $$\{N_1N_2N_3, N_2N_1N_2, N_3N_2N_1\}$$
  for any $$N_1, N_2, N_3 \in \mathcal {B}$$.
2. *X* can include a maximum of 3 out of 4 codons from the set
  $$\{N_1N_2c(N_2), N_2c(N_2)c(N_1), N_3N_4N_1, c(N_1)N_3N_4\}$$
  for any $$N_1,N_2,N_3,N_4\in \mathcal {B}$$.
3. *X* can include a maximum of 5 out of 6 codons from the set
  $$\{N_1N_2c(N_1), N_1c(N_2)c(N_1), c(N_2)N_2N_1, c(N_1)c(N_2)N_2, N_2N_1N_2, c(N_2)c(N_1)c(N_2)\}$$
  for any $$N_1,N_2\in \mathcal {B}$$.

#### Proof

1. Assume that
  $$N_1N_2N_3, N_2N_1N_2, N_3N_2N_1\in X$$
  Let us now consider the concatenation
  $$N_3N_2N_1N_2N_1N_2.$$
  It has two decompositions on a circle
  $$N_3N_2N_1|N_2N_1N_2\quad \text{ and }\quad N_2N_1N_2|N_1N_2N_3.$$
  This is a contradiction to the circularity of *X*.
2. Assume that
  $$N_1N_2c(N_2), N_2c(N_2)c(N_1), N_3N_4N_1, c(N_1)N_3N_4\in X$$
  Let us now consider the concatenation
  $$N_2c(N_2)c(N_1)N_3N_4N_1.$$
  It has two decompositions on a circle
  $$N_2c(N_2)c(N_1)|N_3N_4N_1\quad \text{ and }\quad N_1N_2c(N_2)|c(N_1)N_3N_4.$$
  This is a contradiction to the circularity of *X*.
3. Assume that
  $$N_1N_2c(N_1), N_1c(N_2)c(N_1), c(N_2)N_2N_1, c(N_1)c(N_2)N_2, N_2N_1N_2, c(N_2)c(N_1)c(N_2)\in X$$
  Let us now consider the concatenation
  $$N_1c(N_2)c(N_1)c(N_2)c(N_1)c(N_2)N_2N_1N_2c(N_1)c(N_2)N_2.$$
  It has two decompositions on a circle
  $$N_1c(N_2)c(N_1)|c(N_2)c(N_1)c(N_2)|N_2N_1N_2|c(N_1)c(N_2)N_2\quad \text{ and }$$
  $$c(N_2)c(N_1)c(N_2)|c(N_1)c(N_2)N_2|N_1N_2c(N_1)|c(N_2)N_2N_1.$$
  This is a contradiction to the circularity of *X*.

These rules apply to any circular code but have particular implications for self-complementary codes. The first rule for example implies that two codon/anticodon pairs of type 2 $N_1N_2c(N_1), N_1c(N_2)c(N_1)$ and $c(N_1)N_2N_1, c(N_1)c(N_2)N_1$ with $N_1\ne N_2, N_1\ne c(N_2)$ exclude two codon/anticodon pairs of type 5

$$N_2N_1N_2/ c(N_2)c(N_1)c(N_2)\quad \text{ and }\quad N_2c(N_1)N_2/ c(N_2)N_1c(N_2)$$

(and by symmetry, one pair of type 2 and one of type 5 exclude another type 2 pair).

## Applications of the tool

In this section, we investigate some properties of self-complementary $C^3$ codes using the online tool to verify or contradict certain hypotheses.

### About extensibility of self-complementary $C^3$-codes to maximum size

Michel et al. in Michel et al. (2016) found that not all self-complementary circular codes can be extended to contain 20 codons. Some codes can be extended to a maximum of 18 codons before excluding all remaining codons. We investigate this question in regard to the $C^3$ property, starting with the hypothesis that all self-complementary $C^3$ codes can be extended to self-complementary $C^3$ codes of maximum size. This hypothesis can be disproved with a simple example constructed using the tool. The following is a self-complementary $C^3$-code of cardinality 10, which cannot be extended to one of the 216 self-complementary $C^3$-codes of maximum size 20:

#### Example 1

$$X=\{TAA, TTA, GCC, GGC, ACT, AGT, CAA, TTG, AGG, CCT\}$$

This code was constructed in the online tool, by selecting "Type: C3", "Self-complementarity: Obey" and "Maximality: Ignore", we can try selecting and deselecting various combinations of codons until an entire row of codons is excluded.

Michel et al., while investigating this question in relation to circular codes (without the $C^3$ property), found (see Michel et al. 2016) that self-complementary 18-trinucleotide circular codes can be maximal, and even 16-trinucleotide circular codes can be dimaximal, meaning no more codon/anticodon pairs can be added without breaking circularity, but a singular codon can be added by itself without breaking circularity (but it does break self-complementarity in the process). We investigated the code tables presented in Michel et al. (2016) (see Tables 1, 2) looking for relationships between the codes and whether or not the codes obey the $C^3$ property. The result of this analysis can be found in the tables below.

**Table 1:** 32 maximum self-complementary circular codes of cardinality 18 according to Michel et al. (2016). Bold labels indicate selfcomplementary C3-codes. The numbering is taken from the article Michel et al. (2016)

Id	Sw	Yr	Km	$\pi _{CG}$	$\pi _{AT}$	$\pi _{ACTG}$	$\pi _{AGTC}$
1	32	16	20	3	28	9	18
2	31	14	19	4	27	10	17
**5**	**29**	**13**	**24**	**11**	**26**	**7**	**23**
6	30	15	22	12	25	8	21

**Table 2:** The class of 32 maximal self-complementary 18-trinucleotide circular codes. This class of codes is also dimaximal Michel et al. (2016)

Label	Codons
1	AAC, AAG, AAT, ACG, ACT, AGT, ATT, CAG, CCG, CGG, CGT, CTG, CTT, GGA, GTT, TCA, TCC, TGA
2	AAC, AAG, AAT, ACG, ATT, CAG, CCG, CGG, CGT, CTA, CTG, CTT, GGA, GTT, TAG, TCA, TCC, TGA
3	AAC, AAG, AAT, ACT, AGC, AGT, ATT, CCA, CTT, GAC, GCC, GCT, GGC, GTC, GTT, TCA, TGA, TGG
4	AAC, AAG, AAT, AGC, ATT, CCA, CTT, GAC, GCC, GCT, GGC, GTA, GTC, GTT, TAC, TCA, TGA, TGG
5	AAC, AAT, ACG, ACT, AGA, AGC, AGT, ATT, CCG, CGG, CGT, GCT, GGA, GTT, TCA, TCC, TCT, TGA
6	AAC, AAT, ACG, ACT, AGA, AGT, ATT, CAG, CCG, CGG, CGT, CTG, GGA, GTT, TCA, TCC, TCT, TGA
7	AAC, AGG, ATG, CAC, CAG, CAT, CCG, CCT, CGG, CTA, CTG, GAC, GTC, GTG, GTT, TAA, TAG, TTA
8	AAC, AGG, CAC, CAG, CCG, CCT, CGG, CTA, CTG, GAC, GTC, GTG, GTT, TAA, TAG, TCA, TGA, TTA
9	AAC, AGG, CAG, CCA, CCG, CCT, CGG, CTA, CTG, GAC, GTC, GTT, TAA, TAG, TCA, TGA, TGG, TTA
10	AAC, AGG, CCA, CCG, CCT, CGG, CTA, GAC, GCA, GTC, GTT, TAA, TAG, TCA, TGA, TGC, TGG, TTA
11	AAG, AAT, ACA, ACG, ACT, AGC, AGT, ATT, CCA, CGT, CTT, GCC, GCT, GGC, TCA, TGA, TGG, TGT
12	AAG, AAT, ACA, ACT, AGC, AGT, ATT, CCA, CTT, GAC, GCC, GCT, GGC, GTC, TCA, TGA, TGG, TGT
13	AAG, ACC, ATC, CAG, CTC, CTG, CTT, GAC, GAG, GAT, GCC, GGC, GGT, GTA, GTC, TAA, TAC, TTA
14	AAG, ACC, CAG, CGA, CTG, CTT, GCC, GGA, GGC, GGT, GTA, TAA, TAC, TCA, TCC, TCG, TGA, TTA
15	AAG, ACC, CAG, CTC, CTG, CTT, GAC, GAG, GCC, GGC, GGT, GTA, GTC, TAA, TAC, TCA, TGA, TTA
16	AAG, ACC, CAG, CTG, CTT, GAC, GCC, GGA, GGC, GGT, GTA, GTC, TAA, TAC, TCA, TCC, TGA, TTA
17	AAT, ACC, ACG, ACT, AGT, ATC, ATT, CAA, CAG, CGT, CTG, GAT, GCC, GGA, GGC, GGT, TCC, TTG
18	AAT, ACC, ACT, AGT, ATC, ATT, CAA, CAG, CTG, GAC, GAT, GCC, GGA, GGC, GGT, GTC, TCC, TTG
19	AAT, ACT, AGC, AGG, AGT, ATG, ATT, CAT, CCA, CCG, CCT, CGG, GAA, GAC, GCT, GTC, TGG, TTC
20	AAT, ACT, AGG, AGT, ATG, ATT, CAG, CAT, CCA, CCG, CCT, CGG, CTG, GAA, GAC, GTC, TGG, TTC
21	AAT, ACT, AGT, ATC, ATT, CAA, CAC, CAG, CTG, GAC, GAT, GCC, GGA, GGC, GTC, GTG, TCC, TTG
22	AAT, ACT, AGT, ATG, ATT, CAG, CAT, CCA, CCG, CGG, CTC, CTG, GAA, GAC, GAG, GTC, TGG, TTC
23	AAT, ATC, ATT, CAA, CAC, CAG, CTG, GAC, GAT, GCC, GGA, GGC, GTA, GTC, GTG, TAC, TCC, TTG
24	AAT, ATG, ATT, CAG, CAT, CCA, CCG, CGG, CTA, CTC, CTG, GAA, GAC, GAG, GTC, TAG, TGG, TTC
25	ACA, ACC, ACT, AGT, CAG, CCG, CGA, CGG, CTG, GAA, GGT, TAA, TCA, TCG, TGA, TGT, TTA, TTC
26	ACA, ACC, ACT, AGT, CCG, CGA, CGG, GAA, GCA, GGT, TAA, TCA, TCG, TGA, TGC, TGT, TTA, TTC
27	ACC, ACT, AGT, ATG, CAA, CAG, CAT, CCG, CGA, CGG, CTG, GAA, GGT, TAA, TCG, TTA, TTC, TTG
28	ACC, ACT, AGT, CAA, CAG, CCG, CGA, CGG, CTG, GAA, GGT, TAA, TCA, TCG, TGA, TTA, TTC, TTG
29	ACT, AGA, AGG, AGT, CAA, CCT, CGA, GCA, GCC, GGC, TAA, TCA, TCG, TCT, TGA, TGC, TTA, TTG
30	ACT, AGA, AGG, AGT, CAA, CCT, GAC, GCA, GCC, GGC, GTC, TAA, TCA, TCT, TGA, TGC, TTA, TTG
31	ACT, AGG, AGT, ATC, CAA, CCT, GAA, GAC, GAT, GCA, GCC, GGC, GTC, TAA, TGC, TTA, TTC, TTG
32	ACT, AGG, AGT, CAA, CCT, GAA, GAC, GCA, GCC, GGC, GTC, TAA, TCA, TGA, TGC, TTA, TTC, TTG

Of a total of 32 maximum self-complementary circular codes of cardinality 18, only 8 have the $C^3$ property. All these codes belong to the same equivalence class according to the action of the group *L* (see Fimmel et al. 2015), i.e. all the codes are subject to the permutations of the self-complementarity preserving subgroup *L* of the symmetric group $S_{\mathcal {B}}$:

Bold labels indicate self-complementary $C^3$-codes. The numbering is taken from the article Michel et al. (2016).

The complete list of codes with the corresponding numbers can be found in Table 2 for the convenience of the reader:

The code from the Example 1 can be extended to 4 self-complementary circular codes of size 18, namely the codes labelled 29, 30, 31, 32 from Table 2. One of those codes (29) also has the $C^3$ property (see Table 1).

### Why are there 216 self-complementary $C^3$ codes of maximum size?

In this section, we investigate why there are exactly 216 maximal self-complementary $C^3$-codes and how they can be constructed. We do not succeed in a purely theoretical proof and construction principle but are able to give a solid theoretical approach. We start with the following observation that determines how many codon/anticodon pairs of type 3 can be contained in one of the self-complementary $C^3$-codes of maximum size.

#### Lemma 3

Let $x_1, \cdots , x_4$ be codons of type 3. Then the set

$$X=\{x_1, \cdots , x_4, \overset{\longleftarrow }{c(x_1)}, \cdots , \overset{\longleftarrow }{c(x_4)}\ \}$$

is not circular. In particular, any self-complementary $C^3$-code of maximum size can contain at most 3 codon/anticodon pairs of type 3.

#### Proof

Assume that $x_1, \cdots , x_4$ are codons of type 3 and assume that *X* is circular. Hence they are of the form $x_i=N_iM_ic(M_i)$ with $N_i\not = M_i$ and $M_i \not = c(N_i)$. Thus for any choice of $N \in \mathcal {B}$ there can be at most 2 out of the 4 $x_i$ with $N_i=N$.

Case 1: $N_i=c(N_j)$ for some $i \not =j \le 4$. Then we have the $x_i=N_iM_ic(M_i)$ and $x_j=N_jM_jc(M_j)=c(N_i)M_jc(M_j)$. Consider the concatenation
  $$x_ix_j=N_iM_ic(M_i)\mid c(N_i)M_jc(M_j)=N_i \mid M_ic(M_i)c(N_i) \mid M_jc(M_j)$$
  However, $M_ic(M_i)c(N_i)= \overset{\longleftarrow }{c(N_iM_ic(M_i))}= \overset{\longleftarrow }{c(x_i)}$ and $M_jc(M_j)N_i=M_jc(M_j)c(N_j)= \overset{\longleftarrow }{c(N_jM_jc(M_j))}= \overset{\longleftarrow }{c(x_j)}$ and therefore $x_ix_j$ has two different decompositions contradicting circularity.
Case 2: $N_i \not =c(N_j)$ for all pairs $i \not =j \le 4$. Then there must be two basis $N_1 \not =N_3$ and $N_1 \not =c(N_3)$ such that $x_1, \cdots , x_4$ are of the form
  $$N_1N_3c(N_3), N_1N_4c(N_4), N_3N_1c(N_1), N_3N_2c(N_2)$$
  where $N_2=c(N_1)$ and $N_4=c(N_3)$. For instance *AGC*, *ACG*, *CAT*, *CTA* could be such a situation. Looking at the anticodons, we also get
  $$N_3c(N_3)N_1, N_4c(N_4)c(N_1), N_1c(N_1)c(N_3), N_2c(N_2)c(N_3)$$
  in *X*. We now look at the concatenation
  $$N_1c(N_3)N_3\mid N_2c(N_2)c(N_3) \mid N_3N_1c(N_1) \mid N_4c(N_4)c(N_1)$$
  This has a second decomposition, namely
  $$N_1 \mid c(N_3)N_3N_2 \mid c(N_2)c(N_3)N_3 \mid N_1c(N_1)N_4 \mid c(N_4)c(N_1)=$$
  $$c(N_2) \mid N_4c(N_4)c(N_1) \mid N_1c(N_3)N_3 \mid N_1c(N_1)c(N_3) \mid N_3N_2$$
  again contradicting circularity.

We also have the following result

#### Lemma 4

The four codon/anticodon pairs of type 2 together form a non-circular code.

#### Proof

By definition the four codon/anticodon pairs of type 2 are

$$ACT,AGT,TCA,TGA,CAG,CTG,GAC,GTC$$

Clearly $ACT \mid GTC = A \mid CTG \mid TC$ has two decompositions contradicting circularity. $\square$

As a corollary we obtain

#### Corollary 1

Any self-complementary $C^3$-code of maximum size contains at least one and at most 3 codon/anticodon pairs of type 3.

#### Proof

This follows directly from the Lemmas 3 and 4. $\square$

Hence, in order to calculate the number of self-complementary $C^3$-codes of maximum size (and in order to construct them) we follow the strategy to start with either exactly 1, 2 or 3 codon/anticodon pairs of type 3 and to understand what codon/anticodon pairs of type 2, respectively, type 1 can be added to obtain a self-complementary $C^3$-code. Another easy lemma is needed.

#### Lemma 5

The following holds:

1. If a codon *x* is of type 0 or 1, then its cyclic shifts are again either of type 0 or type 3.
2. If a codon *x* is of type 2 or 3, then its cyclic shifts are again either of type 2 or type 3.
3. If a codon *x* is of type 4 or 5, then its cyclic shifts are again either of type 4 or type 5.

Moreover, a choice of three representatives from equivalence classes of type 3 codons completely determines the choice of representatives from equivalence classes of type 1 codons if the union is a self-complementary $C^3$-code.

#### Proof

Clear by Definition of types and the Theorem 2 points (2) and (5). $\square$

We can now show that there are exactly 216 maximal self-complementary $C^3$-codes.

#### Theorem 5

There are exactly 216 self-complementary $C^3$-codes.

#### Proof

By the above Corollary 1, we know that any self-complementary $C^3$-code of maximum size must contain 1, 2 or 3 codon/anticodon pairs of type 3. Moreover, note that any self-complementary $C^3$-code of maximum size must contain exactly one codon/anticodon pair of each row in the type table displayed, e.g. in Figure 7 since the rows match the equivalence classes of codons under circular shifts (not under the operation of the group L). For the convenience of the reader, we include the type table again in order to illustrate the proof.
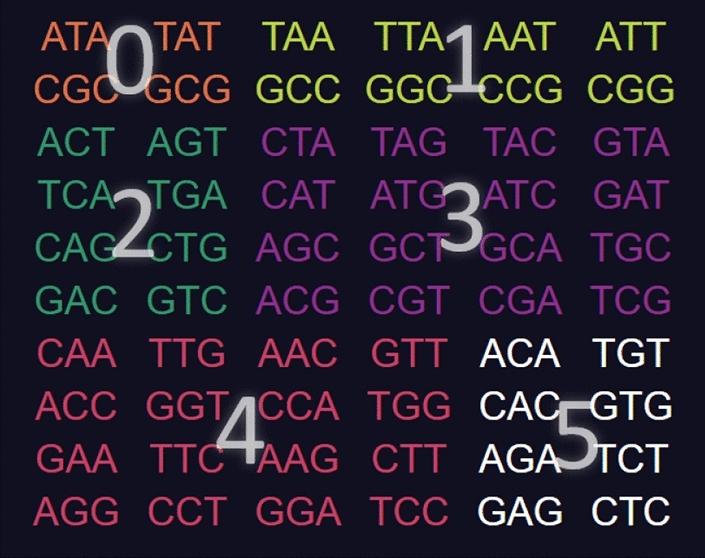


Thus, one has to determine how many possibilities there are to choose exactly one codon/anticodon pair from each row in the type table without obtaining a contradiction to the $C^3$-property. In the sequel equivalence class will always mean with respect to cyclic shifts and not under *L* unless explicitly stated.

1. *Case 1 - exactly 1 codon/anticodon pair of type 3:* Now there are exactly 8 possibilities for the case of exactly one type 3 codon/anticodon pair. All of these must be symmetric since they form one equivalence class under *L* (compare Lemmas 1 and 2). Thus we choose one type 3 codon/anticodon pair with our codon/anticodon selector tool. Due to the maximum size of the code, this implies that there should be 3 codon/anticodon pairs of type 2 in the code (from the remaining green/purple rows in the type table). It is then easy to see with our tool that the selection of the 3 codon/anticodon pairs of type 2 and one pair of type 3 determines uniquely the selection of only one codon/anticodon pair from each equivalence class of type 1 and from two equivalence classes of types 4 and 5. This leaves a choice of two pairs each from the two remaining cyclic equivalence classes of types 4 (note that a cyclic equivalence class of type codons may also include codons of type 5). Hence 4 codes are therefore possible in total with this choice. This gives $4*8=32$ such codes.
2. *Case 2 - exactly 2 codon/anticodon pairs of type 3:* For exactly two codon/anticodon pairs of type 3, we have again 8 choices (and not $$28=\left( {\begin{array}{c}8\\ 2\end{array}}\right)$$): Select the codon/anticodon pair of type 3 $N_1N_2c(N_2)/N_2c(N_2)c(N_1)$. Using our tool, it is easy to see that it can only be combined with the pairs $N_1c(N_2)N_2/c(N_2) N_2c(N_1)$ or $N_1c(N_1)c(N_2)/N_2 N_1c(N_1)$. Otherwise, 3 codon/anticodon pairs of type 2 are excluded at once, forcing the inclusion of the third codon/anticodon pair of type 3 in the code (and this will be considered in the next case).
  Depending on which of the two pairs we choose, we get two groups:
  - $$\{CTA, TAG, CAT, ATG\}, \{TAC, GTA, ATC, GAT\}, \{AGC, GCT, ACG, CGT\}, \{GCA, TGC, CGA, TCG\}$$
  - $$\{CTA, TAG, CGA, TCG\}, \{ATC, GAT, AGC, GCT\}, \{TAC, GTA, GCA, TGC\}, \{CAT, ATG, ACG, CGT\}$$
  Every combinations of two pairs of type 3 in a group can be mapped to another combination in the same group (including itself) using the action of the *L* group (compare Lemmas 1 and 2). It is therefore sufficient to determine the number of possible codes for one combination from each group. Using the codon/anticodon selector every choice leads to 14 codes. This gives $14*4+14*4=112$ such codes.
3. *Case 3 - exactly 3 codon/anticodon pairs of type 3:* Again, also for exactly three codon/anticodon pairs of type 3 we have exactly 8 choices: Select the codon/anticodon pair of type 3 $N_1N_2c(N_2)/N_2c(N_2)c(N_1)$. With this selection, two further codon/anticodon pairs of type 3 are excluded according to Theorem 2 point (5), namely $c(N_2)N_2N_1/c(N_1) c(N_2)N_1$ (reversed pair) and $c(N_2)c(N_1)N_1/c(N_1)N_1N_2$.
  Since a total of 3 codon/anticodon pairs of type 3 must be selected, at least one of them must be selected from one of the two non-complete cyclic equivalence classes $[c(N_2)c(N_1)N_1/c(N_1)N_1N_2]$ or $[c(N_2)N_2N_1/c(N_1) c(N_2)N_1]$. By choosing the only possible pair of type 3 from the cyclic equivalence class $[c(N_2)c(N_1)N_1/c(N_1)N_1N_2]$, the remaining pair of type 3 from the cyclic equivalence class $[c(N_2)N_2N_1/c(N_1) c(N_2)N_1]$ and one pair of type 3 from the complete remaining cyclic equivalence class are excluded.
  With the choice of the only possible pair of type 3 from the cyclic equivalence class $[c(N_2)N_2N_1/c(N_1) c(N_2)N_1]$, the remaining pair of type 3 from the cyclic equivalence class $[c(N_2)c(N_1)N_1/c(N_1)N_1N_2]$ is excluded, while the equivalence class that remained complete still offers 2 choices.
  We therefore have a total of 8*(1+2)/3=8 choices. To be precise, the combinations in question are:
  $$\{CAT, ATG, AGC, GCT, ACG, CGT\}, \{ATC, GAT, AGC, GCT, ACG, CGT\}, \{CTA, TAG, GCA, TGC, CGA, TCG\}, \{TAC, GTA, GCA, TGC, CGA, TCG\}, \{CTA, TAG, CAT, ATG, ACG, CGT\}, \{CTA, TAG, CAT, ATG, CGA, TCG\}, \{TAC, GTA, ATC, GAT, AGC, GCT\}, \{TAC, GTA, ATC, GAT, GCA, TGC\}$$
  All possible combinations of three pairs of type 3 can be mapped to each other using the action of the *L* group (compare Lemmas 1 and 2). It is therefore sufficient to determine the number of possible codes for one selection only. Every choice leads to 9 codes. This gives $9*8=72$ such codes.

So in total we get $4*8+8*9+8*14=32+72+112=216$ maximal self-complementary $C^3$-codes. $\square$

## Conclusions

In this paper, we analyse three classes of error-detecting codes, comma-free, self-complementary circular and self-complementary $C^3$-codes, with respect to the question which codon combinations are excluded in such codes. For this purpose, a computer aid tool was developed to better identify existing patterns. The results were then formally proved mathematically. The results of the analysis were used to explain the number of self-complementary $C^3$-codes of maximum size (Sect. 5.2). Another question that was answered was whether each self-complementary $C^3$-code can be extended to a self-complementary $C^3$-code of maximum size (Sect. 5.1).

## Data Availability

All data generated or analysed during this study are included in this published article.

## Footnotes

1 “Only excludes” means that the codon/anticodon pairs mentioned cannot be together in a self-complementary $C^3$ code. For all combinations with the other codon/anticodon pairs, there is a self-complementary $C^3$-code that contains them.
